# Mobile element scanning (ME-Scan) by targeted high-throughput sequencing

**DOI:** 10.1186/1471-2164-11-410

**Published:** 2010-06-30

**Authors:** David J Witherspoon, Jinchuan Xing, Yuhua Zhang, W Scott Watkins, Mark A Batzer, Lynn B Jorde

**Affiliations:** 1 Dept. of Human Genetics, University of Utah Health Sciences Center, Salt Lake City, Utah, 84112, USA; 2 Dept. of Biological Sciences, Louisiana State University, Baton Rouge, Louisiana, 70803, USA

## Abstract

**Background:**

Mobile elements (MEs) are diverse, common and dynamic inhabitants of nearly all genomes. ME transposition generates a steady stream of polymorphic genetic markers, deleterious and adaptive mutations, and substrates for further genomic rearrangements. Research on the impacts, population dynamics, and evolution of MEs is constrained by the difficulty of ascertaining rare polymorphic ME insertions that occur against a large background of pre-existing fixed elements and then genotyping them in many individuals.

**Results:**

Here we present a novel method for identifying nearly all insertions of a ME subfamily in the whole genomes of multiple individuals and simultaneously genotyping (for presence or absence) those insertions that are variable in the population. We use ME-specific primers to construct DNA libraries that contain the junctions of all ME insertions of the subfamily, with their flanking genomic sequences, from many individuals. Individual-specific "index" sequences are designed into the oligonucleotide adapters used to construct the individual libraries. These libraries are then pooled and sequenced using a ME-specific sequencing primer. Mobile element insertion loci of the target subfamily are uniquely identified by their junction sequence, and all insertion junctions are linked to their individual libraries by the corresponding index sequence. To test this method's feasibility, we apply it to the human *AluYb8 *and *AluYb9 *subfamilies. In four individuals, we identified a total of 2,758 *AluYb8 *and *AluYb9 *insertions, including nearly all those that are present in the reference genome, as well as 487 that are not. Index counts show the sequenced products from each sample reflect the intended proportions to within 1%. At a sequencing depth of 355,000 paired reads per sample, the sensitivity and specificity of ME-Scan are both approximately 95%.

**Conclusions:**

Mobile Element Scanning (ME-Scan) is an efficient method for quickly genotyping mobile element insertions with very high sensitivity and specificity. In light of recent improvements to high-throughput sequencing technology, it should be possible to employ ME-Scan to genotype insertions of almost any mobile element family in many individuals from any species.

## Background

Mobile elements (MEs) are DNA sequences that can replicate and insert themselves into new loci within larger host genomes. This strategy has proved very successful: MEs are evolutionarily ancient, highly diversified in form, ubiquitous in distribution, and often extremely numerous within their host populations [1]. Whole genome sequencing of representatives of many species has allowed great insight into the diversity, number and deep evolutionary history of MEs [2], but it provides very limited information about the frequencies and distributions of ME insertions in populations.

Much of our knowledge about the patterns of ME insertion variation, especially in humans, has been collected by first ascertaining polymorphic ME insertion loci in a small sample of chromosomes and then genotyping those loci in a larger sample from the population (*e.g*., [3-5]). In some cases, loci that contain insertions of recently-active MEs are identified in publicly available DNA sequences (such as the first human genome sequence), then screened by PCR and gel electrophoresis in a small number of individuals to identify the loci that are polymorphic (*e.g*., [6-13]). In other cases, polymorphic loci are identified by comparing publicly available DNA sequences from multiple individuals (such as whole genomes and sequences generated for surveys of genetic variation; [14-16]). The PCR-based locus-by-locus genotyping approach is labor intensive when many loci and individuals are being studied. Moreover, these approaches are limited to identifying and studying polymorphisms that are common in the populations and genomic regions that are best represented in public sequence databases, which introduces ascertainment biases into the data. Since DNA sequences in public databases are collected by heterogeneous methods for various purposes, it is difficult to quantitatively model those ascertainment biases, and investigations that require specific sampling designs (*e.g*., pedigrees) are precluded. Current full-genome sequencing methods remain prohibitively expensive for studies of population variation, and it is difficult to reliably identify indels of any kind in low-coverage genomes assembled from short reads. Methods that rely on subtractive hybridization [5,17,18] allow researchers to efficiently ascertain polymorphic insertion loci in population samples of their own design, but the number of loci that can be identified using these methods has been limited by the number of samples that can be processed simultaneously and the effort required to confirm candidate loci by cloning and sequencing.

Transposon Display (TD) methods [19-25] can generate presence/absence genotypes for a subset of ME insertion loci in all members of a sample. TD avoids ascertainment bias for common insertions, since the specific subset of loci that are genotyped is determined by the choice of restriction enzymes and design of PCR primers, not by the frequencies of the insertions. The number of loci that can be genotyped at one time is limited by the requirement that each insertion be identifiable as a unique, reliably distinguishable band on a polyacrylamide gel. Since insertions at both fixed and variable loci generate bands, large numbers of fixed "background" insertions (as with human *Alu *elements) can greatly reduce the useful genotyping capacity of a TD experiment. In order to confirm results or compare them across methods, it is typically necessary to dissect PCR products out of bands on TD gels for reamplification, cloning and sequencing.

Here we describe a cost-effective method for accurately and quickly identifying nearly all insertions of a given mobile element family in every individual of a large sample. Mobile Element Scanning (ME-Scanning) relies on targeted high-throughput sequencing to efficiently read nearly all the junctions between insertions of a class of mobile elements and the genomic flanks of those insertions. High target specificity (and thus efficiency) is achieved by using element-specific PCR and sequencing primers to amplify and sequence only the desired element-flank junctions. Each mobile element insertion is uniquely identified by its precise insertion position and the flanking genomic sequence, and the presence or absence of that junction sequence in sequence reads derived from an individual DNA sample indicates the presence or absence of the insertion in that individual. To make efficient use of the capacity of high-throughput sequencing platforms, each individual sample is labeled with a unique 5-bp index, and multiple samples are pooled together for sequencing (see, *e.g*., [26-35]).

The *AluYb8 *and *AluYb9 *retrotransposon subfamilies (*AluYb8/9 *henceforth) in the human genome provide an interesting and challenging target for testing ME-Scan. Due to the recent retrotranspositional activity of the *AluYb8/9 *subfamily, there are thousands of insertion loci to be assayed, including many polymorphic ones [8,36]. The long history of *Alu *retrotransposition in primates has created a background of nearly one million older *Alu *copies [37] that must be avoided in order to assay *AluYb8/9 *insertions alone. Finally, the human genome reference sequence [37] and previous *Alu*-genotyping studies [4] provide independent information against which we can measure the performance of ME-Scan. In this study, we demonstrate that ME-Scan makes efficient use of sequencing output; that pooled libraries are evenly represented in the sequencing results; and that we are able to identify *AluYb8/9 *insertions with a sensitivity and specificity of ~95% (using a sequencing depth of 355,000 paired reads per sample.)

## Results

### Overview of ME-Scan

**Figure 1:**
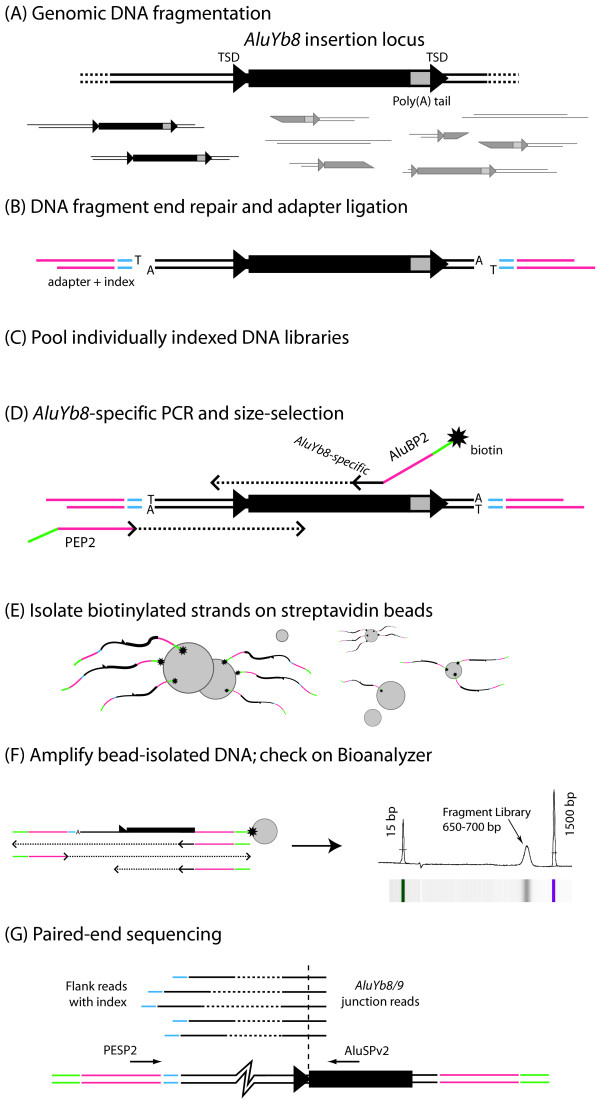
**Mobile Element Scanning (ME-Scan) Library Preparation and Sequencing Protocol**. (A) dsDNA genomic DNA is extracted and then fragmented by sonication. An *AluYb8/9 *element is depicted (black rectangle: *Alu *element; gray box: Poly-A tail of the *Alu*; TSD: target site duplication). Some fragments (darker) will contain most or all of the element along with some upstream genomic sequence. (B) Fragment ends are repaired, 3'A overhangs are added, and oligonucleotide adapters (pink) carrying sample-specific indexes (blue) are ligated onto the ends. (C) Multiple indexed libraries are pooled for subsequent processing. (D) A limited number of PCR cycles are performed using a biotinylated *AluYb8/9*-specific PCR primer (ALUBP2) and a primer (PEP2) that anneals to the adapters. PCR products in the 650-700 bp size range are selected using gel electrophoresis. (E) The biotinylated strands are purified away from other products using streptavidin-coated paramagnetic beads. (F) The biotinylated strands are amplified by PCR with primers matching the adapter sequences. The resulting product is checked using an Agilent Bioanalyzer DNA 1000 assay (electropherogram and gel-like image shown.) (G) Paired-end, 2x36-bp sequencing is carried out on the *AluYb8/9*-specific pooled fragment library using a custom *Alu*-specific primer (ALUSPv2) for the first (*Alu *junction) read and the standard adapter-specific primer (PESP2) for the second (genomic flank) read. The junction read begins inside the *Alu *element, yielding 16 bp of *Alu *sequence followed by 20 bp of genomic flank sequence. The flank read contains the 5-bp index and the 'T' added during sample preparation, followed by 30 bp of genomic sequence. Multiple read pairs are depicted, corresponding to different fragments carrying the same *AluYb8/9 *insertion (generic fragment diagrammed at bottom.)

The key steps of the ME-Scan protocol are illustrated in Figure 1, described below, and detailed in Methods. It combines and adapts established methods of Transposon Display [19,20,38], high-throughput sequencing [39], and sample indexing [26-35]. Genomic DNA (Figure 1A) is sheared by sonication. The resulting double-stranded DNA fragments are repaired to blunt ends, and unpaired adenosines are added to the 3' ends. Oligonucleotide adapters with an unpaired 3' thymine are ligated to those fragments (Figure 1B). The adapters contain a 5-bp "index" sequence that is unique to each individual sample. This index is later sequenced and links each read pair with the individual from whom it is derived, so samples can be pooled (Figure 1C) for subsequent steps. At this point, every sequence from every individual's genome is represented by many dsDNA fragments.

To extract only those fragments that contain an *AluYb8/9*, we perform PCR with a biotinylated primer that anneals to a site found only in *AluYb8*/9 elements and a primer that matches the adapter sequence (Figure 1D). This PCR generates some molecules that contain only a fragment of an *Alu *element and no flanking genomic sequence. Size selection by gel electrophoresis is used to enrich the resulting PCR product for molecules that contain most of an *Alu *insertion as well as 50-300 bp of upstream flanking genomic sequence. The biotinylated strands are then bound to streptavidin-coated paramagnetic beads and purified away from nonspecific DNA by magnetic separation (Figure 1E). The product is re-amplified and checked for quality and quantity (Figure 1F). The resulting *AluYb8*-specific DNA fragment library is subjected to paired-end sequencing (2 × 36 bp reads) on one flow cell lane of an Illumina Genome Analyzer II (GAII; Figure 1G). Instead of using Illumina's standard primer for the first sequencing read, we use a custom sequencing primer that anneals to a site 16 bp inside the 5' end of the consensus *AluYb8/9 *sequence. Thus the first read (the "*Alu *junction" read) will typically consist of 16 bp of *AluYb8/9 *sequence followed by 20 bp of genomic sequence. The second-end read (the "genomic flank" read) extends from Illumina's standard primer and will contain a 5-bp individual-specific index, a 'T', and 30 bp of genomic sequence.

**Figure 2:**
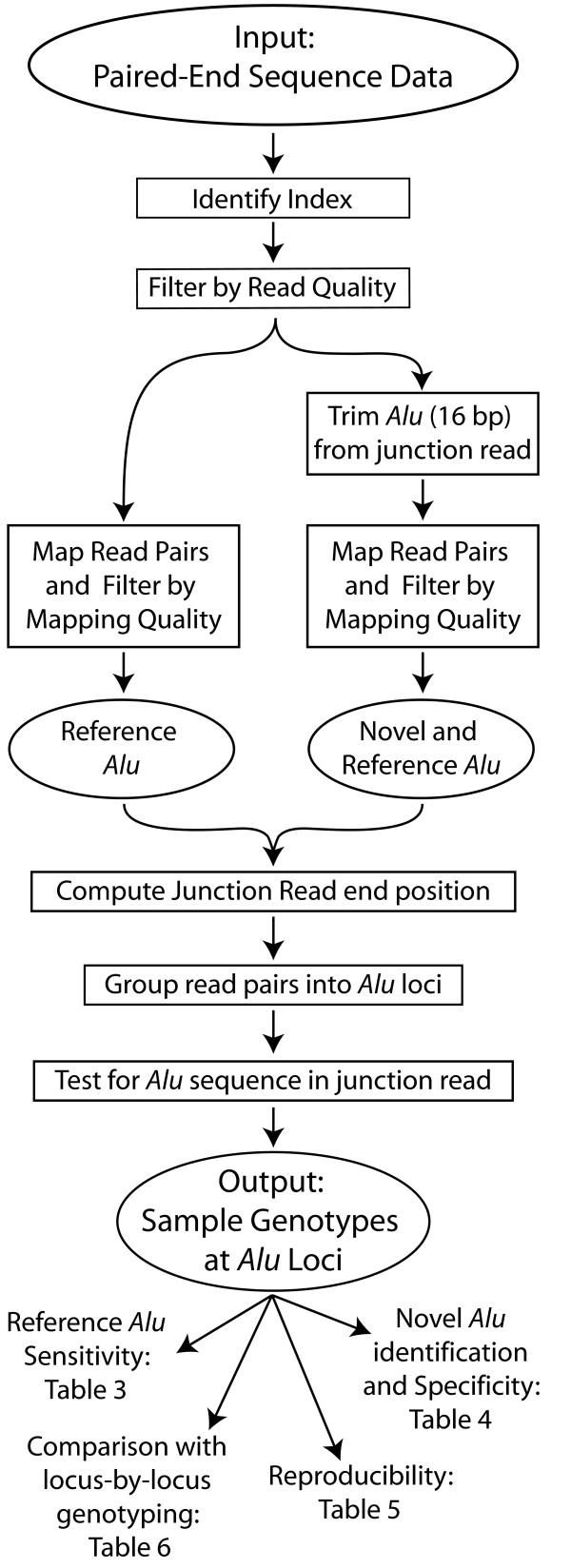
**Sequence analysis pipeline**. Paired-end 2x36-bp sequence reads generated on an Illumina GAII are received as fastq-formatted text files. The index of each read pair is identified and trimmed from the genomic flank reads. Read pairs that could not be assigned a valid index are filtered out. Read quality filtering then removes read pairs in which either read is composed mainly of a single nucleotide, contains too many 'N' base calls, or contains sequences derived from the adapter oligonucleotides. The remaining read pairs are then mapped to the human reference genome, in two ways: first with the expected 16 bp of *Alu *sequence in the junction read, to identify *Alu *insertions that are present in the reference; and then with those 16 bp trimmed off of the junction read, to enable identification of new *Alu *insertions. Read pairs that do not map to a unique location with the proper orientation and the expected distance between them are then filtered out. For each read pair, the position (in the reference genome) of the final nucleotide of the *Alu *junction read is computed for use as the unique identifier of the corresponding insertion. Read pairs are then grouped according to those positional identifiers. Loci that lack *Alu *sequence in the first 16 bp of the junction read are annotated as such and rejected as unreliable. The final data set consists of a list of insertion loci observed in at least one sample and the number of read pairs supporting the presence of each insertion in each indexed sample.

Figure 2 outlines the sequence analysis pipeline. The indexes are identified and stored for every read pair, then trimmed from the flank reads in preparation for mapping. Read pairs that cannot be assigned to a valid individual-specific index, or in which either read is of low quality, are dropped from further analysis. The remaining read pairs are then mapped to the human reference genome sequence both with and without the first 16 bp of the *Alu *junction read, since those 16 bp are expected to consist of *AluYb8/9 *sequence. Poorly mapping read pairs (as defined by BWA [40]; see Methods) are excluded from further analysis. Read pairs that map well with the *Alu *sequence intact represent *AluYb8/9 *insertions that are present in the reference genome. Read pairs that only map well with the *Alu *sequence trimmed off represent *AluYb8/9 *insertions that are absent from the reference. In either case, the position of the last base of the *Alu *junction read is calculated and used as an identifier for that locus. Read pairs are then grouped according to these chromosomal position identifiers, resulting in a list of *Alu *insertion loci identified across all samples. Since loci that lack the expected *Alu *sequence in the supporting junction reads could be non-*Alu *artifacts, they are dropped from further analysis. Loci supported by fewer than 10 read pairs (across all samples and both experiments) are likely to be unreliable and are also dropped from the data set. For the remaining loci, the number of reads corresponding to a particular *Alu *locus and sample index constitutes the evidence for an insertion at that locus in the indexed individual.

### Application of ME-Scan to human *AluYb8/9 *elements

#### Yield of usable sequence data

Table 1 shows the numbers of read pairs obtained for two sequencing experiments as well as the numbers that met our quality criteria (defined in Methods). We loaded the flow cell lanes conservatively in these experiments, so the number of read pairs obtained (2.3 - 3.0 million) is modest relative to the typical capacity of a Genome Analyzer flow cell lane (~8 million read pairs per lane at that time). The yield of high-quality sequences with recognizable indexes exceeds 95%. More than 90% of all read pairs also contain recognizable *AluY *sequence at the beginning of the first read, as expected. Thus very little sequencing capacity is wasted on unusable reads or on fragments that lack *Alu *insertions. Another 14-19% of reads are discarded during read mapping and subsequent analysis. This is due to reads that mapped into low-complexity or repetitive DNA (yielding a BWA map quality of zero), reads that required excessive gaps or mismatches in their alignments to the reference, and reads that mapped to loci that received support from fewer than 10 read pairs across all experiment (see Methods). Nonetheless, at the end of the analysis pipeline, three-quarters of all sequencing reads serve as evidence for specific *Alu *insertions. The *Alu *loci identified and the genotypes generated by ME-Scan are reported in Additional Files 1 and 2, respectively. Of the 5,053 *Alu *loci identified, 2,271 are annotated as *AluYb8/Yb9 *in the human reference genome (hg19/GRCh37), 2,295 are annotated as *Alu *insertions of some other subfamily, and 487 insertions are not present in the human reference genome and therefore probably represent *AluYb8/9 *insertions that are variable in humans. Of the 363 polymorphic *AluYb8/9 *insertion loci listed in dbRIP [16], 223 were identified by ME-Scan in our sample.

**Table 1:** Quantity and quality of paired-end sequencing reads

	Replication experiment		Pooling experiment	
Quality classification	# read pairs	% of total	# read pairs	% of total
Total read pairs	3,047,279	100	2,389,900	100
Both reads of high quality (not a, b or c, below)	2,998,412	98.4	2,315,412	96.9
(a) Either read is > 85% any one base	1,370	0.0450	1,628	0.0681
(b) Either read has > 2 'N' base calls	4,345	0.143	3,073	0.13
(c) Adapter sequence detected in either read	43,192	1.42	69,827	2.9
Both reads high quality, index valid	2,963,735	97.3	2,287,571	95.7
Junction read has *Alu *sequence; both reads high quality; index valid	2,874,329	94.3	2,201,101	92.1
Supports an *Alu *insertion in the final results*	2,458,549	80.7	1,753,750	73.4

*Supports one of the 5,053 insertion loci reported in Additional File 1.

#### Experimental Design

**Figure 3:**
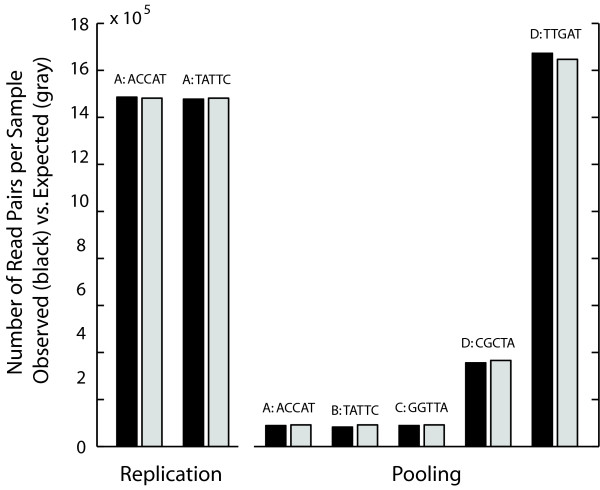
**Observed vs. Expected frequencies of indexes in pooled samples**. Vertical bars represent the numbers of high-quality read pairs that were observed (black), compared with the number expected (white) for each index in two experiments. Expected numbers were calculated from the total numbers of high-quality read pairs for each experiment and the intended pooling proportions (from left to right: 50%, 50% for the Replication experiment; and 4%, 4%, 4%, 16%, 72% for the Pooling experiment). The individuals sampled (A through D) and the index sequences used are shown above the corresponding bars.

We designed two sequencing experiments to test the reproducibility, specificity and sensitivity of ME-Scan at various levels of sequencing coverage (read pairs per sample). DNA samples from four individuals (labeled A through D) were used. For the "Replication" experiment, we processed two aliquots of DNA from individual A in parallel through the ligation step, using a different index for each aliquot, then pooled them in equal amounts for subsequent library preparation and sequencing. For the "Pooling" experiment, we prepared five samples with different indexes (one sample each from individuals A, B and C, and two aliquots from individual D) and combined them in varying proportions (4%, 4%, 4%, 16%, and 72%, respectively) to construct a pooled library. Figure 3 shows that the proportions of each index as recovered by sequencing match the intended proportions to within 1% on average (Table 2). Thus, it should be possible to pool small amounts of DNA from many library preparations without severely under- or over-representing any one library.

**Table 2:** Observed vs. Expected frequencies of indexes in pooled samples

Individual	Index	Read Pairs*	Expected %	Observed %
Replication				
A^1^	ACCAT	1,485,980	50	50.1
A^1^	TATTC	1,477,755	50	49.9
Pooling				
A	ACCAT	88,418	4	3.86
B	TATTC	82,049	4	3.59
C	GGTTA	88,895	4	3.89
D^2^	CGCTA	355,469	16	15.5
D^2^	TTGAT	1,672,740	72	73.1

* Only read pairs passing all QC filters are counted.

^1 ^Two aliquots of a DNA sample from this individual were processed in parallel and pooled for sequencing.

^2 ^A DNA sample from this individual was sonicated, then divided into two aliquots for subsequent library construction steps.

### Sensitivity and Specificity

To estimate the sensitivity of ME-Scan, we searched for false negative results in our data. We first identified a set of presumably fixed *AluYb8/9 *insertions, reasoning that ME-Scan should retrieve all of these insertions in every individual, and that failures to do so would likely represent false negative errors. These insertions are present in the human reference genome assembly, contain good matches to the *AluYb8/9*-specific amplification and sequencing primers we used (with no mismatches in the 3' 10 bp), are of the canonical *AluYb8/9 *size (224 ± 15 bp between the primer annealing sites), and are not known to be polymorphic (*i.e*., are not listed in dbRIP [16,41]). We found 1,708 such loci (Additional File 3). The numbers of these loci that were not retrieved in each sample preparation are shown in Table 3. On the assumption that each of these 1,708 loci is indeed fixed for the insertion in humans, the rate of ME-Scan false negative results ranges from 8% to 25%. The error rate depends on the sequencing effort expended on a sample: the higher the number of reads, the lower the false negative rate.

**Table 3:** Comparison of ME-Scan results to 1,708 presumably fixed AluYb8/9 insertions in the human reference genome

Individual	Index	Number of ME-Scan negative loci	False Negative rate (%)	Genotypes checked by PCR^1^	Checked and Present by PCR^2^
Replication					
A	ACCAT	147	8.61	18	4
A	TATTC	145	8.49	18	4
Pooling					
A	ACCAT	400	23.4	18	4
B	TATTC	420	24.6	18	2
C	GGTTA	400	23.4	17	5
D	CGCTA	164	9.60	17	2
D	TTGAT	134	7.85	17	2
Combined	All	100^3^	5.85	123	23

^1 ^The number of genotypes compared differs from sample to sample due to occasional uncertain genotype calls (missing data) in the PCR and gel genotyping assays.

^2 ^These genotypes represent actual false negative ME-Scan results (absent according to ME-Scan, present by PCR.)

^3 ^For 100 of the 1,708 loci, ME-Scan did not observe the insertion-present allele in any of the samples tested ("Combined").

Out of this set of 1,708 insertion loci, there were 100 loci for which the insertion-present state was not observed by ME-Scan in any of our four samples (Table 3, "Combined"). Thirty-three of these 100 (~2%) are part of recently duplicated regions of the genome (*i.e*., there is at least one 99%-identical copy of the 500 bp sequence upstream of the *Alu *insertion elsewhere in the genome, as detected by BLAST). Read pairs derived from these do not map to a unique position in the genome, and are therefore eliminated by our analysis pipeline. Read pairs corresponding to previously unknown *Alu *insertions into such regions would likewise be eliminated and lead to false negative results. Another 31 of those 100 insertions lack detectable *Alu *sequence in the 16 bp immediately following the sequencing primer binding site. Nearly all of these insertions are in fact observed in the ME-Scan sequencing results. However, in order to filter out artifactual false positives, the ME-Scan pipeline discards loci that lack the expected *Alu *sequence, and so these loci lead to false negative results.

Apart from the loci in recently duplicated regions and those lacking the expected *Alu *sequence in the junction read, it is very likely that some of the 1,708 putatively fixed *AluYb8/9 *elements are in fact not fixed, and are absent in some of our samples. If so, their absences from the ME-Scan results do not represent false negatives. We used PCR and gel electrophoresis to genotype 19 loci that were not in duplicated regions and contained the expected *Alu *sequence in the junction read, but still were not retrieved by ME-Scan in some or all of our samples (see Methods and Additional File 4 for details). The genotypes obtained by PCR and gel-typing for the four individuals are compared with those obtained by ME-Scan in Table 3. In the current ME-Scan analysis pipeline, *Alu *insertions are treated as presence or absence states for purposes of comparison. Of the 19 loci examined, only one had the insertion present in the homozygous state in all four individuals. Four more loci had the insertion present in at least some individuals, but the remaining 14 loci were homozygous for the absence of the insertion in all individuals. Initially, our sensitivity appears to be ~90% at a sequencing coverage of ~355,000 read pairs per sample (164 putative false negatives out of 1,708 loci; Table 3, Pooling experiment, sample indexed with CGCTA.) PCR genotyping suggests that only ~12% (2/17) of the 100 putative false negative genotypes that were not in recently duplicated regions are in fact false negatives, so a better estimate of the number of actual false negative results in this experiment is 76 (33 + 31 + 0.12 × 100), which implies a sensitivity of 95.6%. If we restrict our target set of *Alu *insertions to only those that have intact 5' ends and reside in non-duplicated regions, our sensitivity exceeds 99%.

To estimate the specificity of ME-Scan, we searched for examples of false positive results. Any false positives should be in the set of new *AluYb8/9 *insertions retrieved by ME-Scan, *i.e*., those that we could not annotate based on the reference genome (see Annotation in Methods.) We identified 487 such insertions. We selected 44 loci at random from this set for confirmation by PCR and gel electrophoresis (locus descriptions and PCR primers in Additional File 4). All 44 showed the PCR product expected from the *Alu*-filled allele in at least one sample, indicating that the method is very reliable for detecting new insertion loci. In some individuals, however, an insertion detected by ME-Scan was not detected by PCR and gel electrophoresis, suggesting either that the gel-typing assay was insufficiently sensitive or that there was some cross-contamination between ME-Scan libraries. On the assumption that the ME-Scan results are at fault, the false-positive rate ranged from 3% in the highest-coverage samples to 10% in the lowest-coverage samples (Table 4). The combined biochemical and bioinformatic protocols achieve a specificity of ~96% when based on 355,000 reads per sample (Table 4, sample indexed by CGCTA; 1 out of 27 positive genotypes proved false.) Because all individuals were genotyped by ME-Scan and PCR plus gel electrophoresis for these 44 loci, this data set allows us to examine the rate of false negative ME-Scan results for this class of loci (last two columns of Table 4). No false negatives were observed in the four higher-coverage samples, suggesting that the false negative rate among new *Alu *insertions is as low as that observed for known *AluYb8/9 *loci (above, Table 3.) The data for the three low-coverage samples suggest a higher false-negative rate among new *Alu *insertion loci when relying on lower-coverage sequencing.

**Table 4:** New variable AluYb8/9 loci identified by ME-Scan

Individual	Index	New* *AluYb8/9*	Positive genotypes checked by PCR	False positives	Negative genotypes checked by PCR	False negatives
Replication						
A	ACCAT	259	35	1	9	0
A	TATTC	273	35	1	9	0
Pooling						
A	ACCAT	163	32	3	12	5
B	TATTC	153	20	2	22	3
C	GGTTA	168	19	1	23	5
D	CGCTA	242	27	1	16	0
D	TTGAT	290	27	1	16	0
Combined	All	487	195	10	107	13

* Not observed in the human reference genome (hg19/GRCh37).

### Confirmation of new variable AluYb8/9 insertion loci in additional individuals

In order to test the dimorphic status of loci in the above-described set of 44 new *AluYb8/9 *insertions, we genotyped them by PCR (as above) in a panel of eight additional unrelated individuals: six of east Asian descent (DNA from lymphoblastoid cell lines [42]), one European (J. C. Venter; whole-genome-amplified DNA [15] collected by the J. Craig Venter Institute from blood), and one African individual (NA19376 from Webuye, Kenya; cell line DNA from Coriell cell repository). The genotyping results are reported in Additional File 5. Of the 44 loci, 40 showed the expected *Alu *insertion in at least one individual, confirming that they are dimorphic in humans. Of the loci that lacked the insertion allele in this panel of eight individuals, one did not show the insertion even in the original panel of four individuals (*i.e*., PCR did not confirm the ME-Scan results), and three showed the insertion in only one individual of the four. Any comprehensive survey of genetic variation will identify "singletons" (variants that are observed just once in a population sample), so our results are consistent with that expectation. These three insertions could be rare in humans, they could be limited to a family or just a single individual (*de novo *insertions), or they might be non-heritable insertions resulting from somatic or even cell-line retrotransposition events. In the full set of 487 new *AluYb8/9 *insertions identified by ME-Scan (Table 4), 256 were observed (by ME-Scan) in more than one individual of the original four, which implies that they are polymorphic in humans. The remaining 231 insertions could be rare, *de novo*, or even non-germline insertions. However, 15 of those 231 loci were among the 44 chosen for further genotyping in the panel of 8 additional individuals, and of those 15 loci, 12 (80%) had the insertion present in at least one member of the panel. Thus it is likely that a large majority of the 487 new *AluYb8/9 *loci identified by ME-Scan are in fact polymorphic in humans.

### Reproducibility

In order to study the reproducibility of ME-Scan, we assayed DNA from individual A twice, in parallel and with the same level of coverage ("replication" experiment.) Table 5 shows the results of comparing the *Alu *insertions identified by ME-Scan in the two replicates. Together, the two replicates identified 2,174 *AluYb8/9 *insertions that are known from the reference genome (most are probably fixed in humans; 170 are polymorphisms listed in dbRIP.) Among these, the proportion of insertions that identified in one replicate but not the other (the replication failure rate) is less than 1%. These replicates also recovered 1,390 previously known insertions from *Alu *families other than *AluYb8/9*, but with low reproducibility: each replicate missed more than 400 of the insertions identified in the other. The genome contains hundreds of thousands of *Alu *insertions with poor matches to the amplification and sequencing primers we used, so despite the low probability that any one of them will be amplified and sequenced, we expect to see sporadic amplification of some of these loci in each experiment. By the same token, however, the set of loci that are sporadically amplified will differ from sample to sample, so their reproducibility should be low. The two replicates also yielded 289 new *Alu *insertions (243 of which were common to both replicates), with a replication rate of ~92% in that set (Table 5). This is slightly lower than the ~96% sensitivity observed for the set of known insertions (above.) Perhaps this set of previously unobserved *Alu *insertion loci includes some from other currently active *Alu *families, such as *AluYa5*. These loci may amplify erratically due to their poorly matching primer binding sites, thereby reducing the overall reliability in this set.

**Table 5:** Reproducibility

*Alu *insertion class	Positive loci in either sample*	Absent from ACCAT	Absent from TATTC	Replication failure rate, average %^†^
Known *AluYb8/9*	2,174	20	15	0.805
New variable *Alu*	289	30	16	7.96
Non-specific *Alu*	1,390	434	410	30.4

* Total positive loci are those observed in either of the two samples of the 'replication' experiment, indexed ACCAT and TATTC.

^† ^Number of insertion loci absent in a sample, divided by the total observed in both samples, averaged over the two samples.

### Comparison with previously genotyped AluYb8/9 insertion loci

Numerous researchers, including ourselves, have studied polymorphic *Alu *insertion loci by first ascertaining them in a small set of individuals and then genotyping them in a larger set using locus-specific PCR primers and gel electrophoresis [4,43-45]. We previously used this method to genotype 38 polymorphic *AluYb8/9 *loci in the four individuals that we assayed by ME-Scan in the current work [4] (Additional Files 6 and 7). Table 6 shows the results of comparing the genotypes inferred by both methods for those loci in these individuals. Most of the disagreements between the two methods (11 of 15) are due to negative ME-Scan genotypes in the low-coverage samples (~86,000 reads each; individuals A, B, and C in the Pooling experiment.) It seems likely that these gel-typing results are correct, and that these disagreements are ME-Scan false negatives due to insufficient sequencing depth. The remaining four disagreements involve three replicate ME-Scan genotypes of a single locus in one individual (locus YB8NBC437 on chr13 with *Alu *junction end at position 110,812,488, in individual A) and one genotype at another locus in another individual (locus YB8NBC49 on chr6 with *Alu *junction end at 9,460,373, individual B). ME-Scan indicates the presence of an insertion in these two cases, while previous genotyping indicated homozygous absent genotypes. The consistency of the ME-Scan result for the one locus in individual A suggests that the previous genotyping results might be at fault, so we used PCR and gel electrophoresis to check both genotypes (see Additional file 6 for primers and conditions.) We find that, in fact, both genotypes were heterozygous for the presence of the insertion, in agreement with the ME-Scan results (as noted below Table 6; since we did not systematically re-genotype all these loci, Table 6 reports results of comparisons with the original data.) For this set of loci, given sufficient coverage (*e.g*. 355,000 read pairs per sample), ME-Scan appears to be more accurate than gel typing.

**Table 6:** Comparison of ME-Scan results with previously genotyped AluYb8/9 insertion loci

Individual	Index	ME-Scan Positives	ME-Scan Negatives	False Positives*	False Negatives
Replication					
A	ACCAT	20	11	1	0
A	TATTC	20	11	1	0
Pooling					
A	ACCAT	18	13	1	2
B	TATTC	23	10	0	4
C	GGTTA	23	10	1	5
D	CGCTA	26	7	0	0
D	TTGAT	26	7	0	0
Overall	All	156	69	4	11

* All four of the apparent ME-Scan false positives are due to errors (false negatives) in the previous PCR- and gel-based genotyping.

The HuRef genome sequence of J. Craig Venter [46] offers the opportunity to verify some of the *Alu *insertions we identified that are absent from the hg19/GRCh37 reference. Of 148 HuRef *AluYb8 *and *AluYb9 *insertions of at least 250 bp in length [15], 88 were observed by ME-Scan (*i.e*., their positions are within 50 bp of the computed position of the last base of the *Alu *junction read). Although we have no precise expectation for the number of *AluYb8/9 *insertions that our panel of four East Asian individuals should share with one European (Venter), this result is the equivalent of confirmation by complete sequencing of a sizable set of the previously unobserved loci identified by ME-Scan.

## Discussion

Complete ascertainment and genotyping of *Alu *insertion events in large population samples would give us - for the first time - a clear and panoramic view of their population dynamics. Knowing the genomic distribution of the most recent *Alu *insertions will allow us to decisively disentangle the effects of target site preferences from the effects of natural selection, recombination, and gene conversion that may subsequently alter that distribution [47-50]. From the full site frequency spectrum of insertions, we should be able to estimate the transposition rate and the range of selection coefficients that affect *Alu *insertions [51,52]. It may even be possible to identify factors that cause positive or negative selection on some insertion classes. With sufficient data, we may be able to estimate the evolutionarily relevant, *in vivo *transpositional activities and effects of specific *Alu *elements (*e.g*. "master" elements, suppressors, etc.) by analyzing correlated variation in transposition rates between families and populations.

We have developed a method for quickly, sensitively, and specifically ascertaining nearly all *AluYb8/9 *insertions in several individual samples simultaneously. Other researchers have used similar targeted high-throughput sequencing approaches to identify ME insertions [53,54], but with very different goals. Typically, an engineered ME is used to create many highly uniform insertions of a unique DNA construct in a model organism or cell line. Selection for a phenotype is then applied, and targeted high-throughput sequencing is used to locate a sample of the selected insertions. ME-Scan is designed to identify essentially all members of a class of naturally occurring insertions against a large background of very similar sequences in all members of a population sample.

By using a fraction of the sequencing capacity of two Illumina GAII flow cell lanes to scan four individuals, we have simultaneously identified, mapped, and partially genotyped 487 new variable *AluYb8/9 *insertions in those individuals. Nearly all previously known *AluYb8/9 *insertion loci were also detected. We examined several levels of sequencing effort and found that a level of ~355,000 read pairs per sample allowed the assay to achieve ~95% sensitivity and specificity. Illumina's Genome Analyzer IIx now yields up to 30 million paired sequence reads per flow cell lane [55]. If this read density is approachable with ME-Scan libraries, it will be possible to index and pool 50 individuals per lane with very high sensitivity and specificity.

Even at lower read densities, it should be possible to make more efficient use of the sequencing yield. Theoretically, the number of read pairs per insertion should be Poisson-distributed, with variance equal to the mean coverage. However, in all our samples, the variances are at least an order of magnitude greater than the means. This is a common feature of high-throughput fragment-library sequencing techniques (*e.g*. [28,34,56]). Reducing this overdispersion even moderately would deliver higher performance at lower levels of sequencing effort by reducing the number of read pairs that are effectively wasted on heavily over-represented insertions. It would also increase the number of reads derived from poorly represented insertions that are at risk of being missed altogether. Simple changes to the basic protocol (*e.g*. reducing the number of PCR cycles used, improving the DNA fragmentation method) are likely to reduce that overdispersion.

The length of sequencing reads has increased along with the number of reads per run. It is now possible to obtain paired 100 bp sequence reads instead of the paired 36 bp reads used here [55]. Longer reads would increase both the sensitivity and specificity of the ME-Scan analysis pipeline. By using a sequencing primer that anneals ~30 bp inside the normal 5' end of an *Alu *(instead of 16 bp) and reading 100 bp from that primer, one could obtain more of an *Alu *insertion's sequence (~30 bp) as well as more immediately adjacent genomic sequence (~70 bp). The additional genomic sequence would allow reliable mapping of some read pairs that are now being discarded (*e.g*., reads in regions of low sequence complexity). The additional *Alu *sequence would allow the elimination of spurious non-*Alu *products without discarding evidence of *Alu *insertions with modest 5' truncations. A short second read would still be required to sequence the sample index.

More importantly, using a long (100 bp) junction read would allow construction of a reference-independent analysis pipeline. The ME-Scan analysis pipeline described above depends on mapping reads to a reference genome for two reasons. Firstly, most of our analyses focused on comparing ME-Scan results to the "gold standard" information in the reference genome, so mapping was required in any case. Secondly, the information in paired 36 bp reads is insufficient for highly accurate reference-independent locus identification and genotyping. The first 16 bp of an *Alu *junction read is usually *Alu *sequence, which - being nearly identical for most *Alu *elements - does not distinguish groups of read pairs that represent different insertion loci. The remaining 20 bp (consisting of unique genomic sequence) are often insufficient to clearly distinguish all the groups of reads, since some ambiguity must be allowed to handle sequencing errors and some insertions are in regions of low sequence complexity. When mapping read pairs to a reference, those 20 bp combine with the 30 bp of genomic sequence from the flank read to provide enough information for reliable positioning. This allows read pairs to be grouped according to their mapped location. Unfortunately, since the 30 bp segments of genomic sequence in the flank reads from a particular insertion are derived from sites at varying distances from the junction read, they will not be identical and may not even overlap. Consequently, the flank reads cannot be used to reliably group read pairs based on their sequence alone.

However, with a long *Alu *junction read (*e.g*. 100 bp, with 30 bp derived from the *Alu *element), it should be possible to identify and group all junction reads that represent a particular insertion simply because they will all share nearly identical junction sequences, while reads from different loci will almost always be easily distinguished due to their different sequences (even allowing some tolerance for sequencing errors.) It would no longer be necessary to first map every read pair to a reference genome sequence and then group them into loci by their positions. Genotyping by grouping read pairs according to the near-identity of their junction sequences should deliver accuracy as good as or better than what we have achieved here. In particular, new insertions into one copy of a duplicated genomic segment will not pose a problem. The position of the new insertion within the duplication will be unique, and all reads corresponding to that insertion will group together, just as they would for new insertions into unique regions.

The ability to ascertain and genotype ME insertions without relying on a sequenced reference genome would make ME-Scan very widely applicable: by designing the appropriate amplification and sequencing primers, one could study the population dynamics of nearly any ME family in any organism in detail. The method promises to be robust, quick, reliable and relatively inexpensive: the per-bp cost of sequencing continues to decrease; the indexing and pooling method makes efficient use of high-capacity sequencing platforms; library preparation is technically straightforward; the cost of sample preparation is minimized by the use of sonication rather than nebulization, and by pooling many samples for some processing steps; and the amount of template required has decreased by at least fivefold since we began our experiments [55], further reducing the cost of reagents required for sample preparation. These factors would make a reference-independent ME-Scan method appealing for simultaneously identifying and genotyping polymorphic markers of population genetic diversity in any species.

## Conclusions

ME-Scan enables the rapid, efficient, and highly accurate ascertainment and genotyping (as dominant markers) of thousands of mobile element insertion loci of a particular subfamily in many individuals, even in the presence of a very large background of related elements. With longer sequencing read lengths, it will be possible to apply ME-Scan to species for which no full-genome reference sequence is available. The ability to quickly and inexpensively gather so much detailed information on any ME family in any population represents a breakthrough in the study of the population dynamics and evolution of mobile elements.

## Methods

### Library preparation and sequencing

The steps of the biochemical portion of the ME-Scan method are shown in Figure 1. Seven DNA libraries were prepared using genomic DNA from four unrelated individuals from Vietnam (individual A) or Japan (individuals B, C, and D). All four grandparents of each individual were from the country of origin. DNA was extracted from lymphoblastoid cell lines that were established by Epstein-Barr virus transformation [42]. For library construction, a modified version of the Illumina "Genomic DNA Sample Prep" protocol [39] as used, as follows. Each library was constructed from 5 μg of genomic DNA. Each DNA sample, in 500 μl of TE buffer in a 1.5 ml Eppendorf tubes, was sonicated (Misonix Sonicator 4000, Qsonica, LLC, Newton Connecticut) for 25 s at a 90% duty cycle while suspended in an ice bath (Figure 1A). DNA was then concentrated by centrifugation column (QIAquick spin column), checked by gel electrophoresis, end-repaired, purified, modified to add 3'A overhangs, and column purified again as per the Illumina protocol. Adapter ligation was then performed according to the Illumina protocol, but with custom oligonucleotide adapters based on Illumina's designs (Figure 1B; Illumina oligonucleotide sequences^© ^2006-2008 Illumina, Inc; all rights reserved.) All custom oligonucleotides were synthesized by Integrated DNA Technologies, Inc. (Coalville, Iowa). The oligonucleotides are CPEA1Xa (5' CTC GGC ATT CCT GCT GAA CCG CTC TTC CGA TCT xxx xxT 3', HPLC purified) and CPEA2TruncXa (5' yyy yyA GAT CGG AAG AGC G 3', HPLC purified), where 'x' and 'y' denote the 5 nucleotides and their reverse complements, respectively, that comprise the library-specific indexes or 'barcodes.' See Table 3 for the index sequences used. The ligation products were then purified by column centrifugation (QIAquick) and quantified by spectrophotometry. These seven libraries were pooled (Figure 1C) into two libraries according to the experimental design shown in Table 3 and described in Results. *AluYb8/9*-specific PCR was then carried out on each pooled library (Figure 1D) using a modification of Illumina's protocol ("Enrich the Adapter-Modified DNA Fragments by PCR"; [39]). Instead of Illumina's primers, we used the following: ALUBP2 (5' B-ACA CTC TTT CCC TAC ACG ACG CTC TTC CGA TCT GCC CAG GCC GGA CTG CGG AC 3', 5' biotinylated and gel-purified) and PEP2 (5' CAA GCA GAA GAC GGC ATA CGA GAT CGG TCT CGG CAT TCC TGC TGA ACC GCT CTT CCG ATC T 3', HPLC-purified). These PCR products were purified (QIAquick PCR Purification Kit) and then subjected to size-selection by gel electrophoresis. DNA fragments in the 650-700 bp range were excised and column purified from the excised gel slices ("Purify Ligation Products" [39]). The size-selected DNA was incubated with streptavidin-coated paramagnetic beads (Figure 1E) to first bind the biotinylated ssDNA fragments (those containing *AluYb8/9 *insertions) and then purify them away from non-biotinylated fragments using magnets to immobilize the biotinylated DNA, per the manufacturer's protocol (Dynabeads^® ^MyOne™ Streptavidin C1, Life Technologies, Inc., Carlsbad, California, USA.) The bead-bound DNA was washed into 20 μl of buffer and amplified using the same PCR protocol used above, but with 25 cycles and the standard Illumina Paired-End PCR primers 1 and 2 (PEP1, PEP2). Each library was quantified by Bioanalyzer DNA 1000 chip (Agilent Technologies, Inc., Santa Clara, California, USA) (Figure 1F) and loaded onto a flow cell lane on an Illumina GAII for paired-end sequencing (36 bp sequence on each fragment end; Figure 1G; per manufacturer's recommendations). A custom sequencing primer (ALUSPv2 5' CCC AAA GTG CTG GGA TTA CAG GCG TGA 3', HPLC purified) was used for the first end read instead of Illumina's standard primer.

### Sequence analysis pipeline

The bioinformatic processing steps are outlined in Figure 2. Sequence data was received in two Illumina fastq text files for each experiment ('Replication' and 'Pooling'): one containing the *Alu *junction-end reads and another containing the corresponding genomic flank reads (sequence data available upon request.) Data were handled using an Oracle database [57] and Matlab [58] scripts (available upon request). The 5-bp index for nearly all read pairs could be assigned by simply reading the first 5 bp of the genomic flank read. Where the observed index sequence differed by one nucleotide change from a valid index, the nearest valid index was assigned, except for a small number of sequences (<200) that differed by one nucleotide change from two valid indexes; these read pairs were not assigned an index. Every read was also analyzed for several quality indicators: (1) low complexity (reads consisting of more than 85% one base); (2) excessive 'N' base calls (more than two); and (3) contamination with sequence from the oligonucleotides used to construct the libraries (Smith-Waterman alignment score > 60 when aligned to CPEA1Xa, ALUBP2, PEP2 or their reverse-complements, excluding the 20 bp of *AluYb *sequence in ALUBP2, no gaps allowed). Reads showing any of those defects were flagged as unusable.

BWA [40] was used to map paired reads to the human reference genome. Throughout, we used the UCSC hg19/GRCh37 assembly, without the nine alternate haplotype chromosomes. Mapping proceeds in two steps: BWA first attempts to map all reads independently of each other, and then uses that information in conjunction with expectations about the relative orientation and distance between the two reads in a pair to map all read pairs. The genomic flank reads were mapped after trimming the first 6 bp to remove the index and 'T' added during library construction. The *Alu *junction reads were mapped twice, once with the full 36 bp reads intact, then with the first 16 bp of sequence (which are expected to be part of an *Alu *element) trimmed off, so that the remaining sequence could be mapped even if it was derived from an *Alu *insertion that is absent from the reference genome. We used mostly default BWA options for single-end mapping, with iterative search disabled and allowing a maximum edit distance of two. Two paired-end mapping analyses were then performed to join the flank mapping results with the two versions of the junction mapping results, *i.e*., with the 16 bp of presumed *Alu *sequence intact or trimmed (BWA default options).

Both sets of mapped read pair results were filtered to exclude pairs for which no valid index was identified as well as pairs in which either read was flagged as unusable in the read quality analysis above. We also filtered out read pairs that could not be 'properly mapped' as defined by BWA ('properly mapped' pairs have BWA 'flag' values of 147, 99, 163, 83, indicating read pairs in which both reads mapped uniquely to positions that are within an internally-determined distance of each other, in opposing directions, oriented towards each other). Read pairs in which either read aligned to the reference with more than three mismatched or unmatched bases and read pairs that mapped with a BWA map quality of 0 on either read were discarded. We expect read pairs that represent *AluYb8/9 *elements in the reference sequence to be mapped regardless of whether the 16 bp of *Alu *sequence in the junction read was trimmed or left intact. In such cases, the information gained from mapping the read pair without the 16 bp of *Alu *is redundant and was ignored. Read pairs that were successfully mapped only when the 16 bp *Alu *sequence was trimmed are evidence of new *Alu *insertions.

The set of read pairs that passed the above sequence and mapping quality filters was used to identify *Alu *insertion loci according to their position in the reference genome. The position of the last base pair in the *Alu *junction end read (20 bp past the end of a typical *Alu *insertion) was used as a specific and reliable locus identifier, since that position is present in the reference even if the *Alu *insertion is not. This position was calculated for every mapped read pair, taking into account insertions, deletions and mismatches in the BWA alignment (using information in the 'CIGAR' string) and whether or not the 16 bp of *Alu *sequence was included in the mapped read. Read pairs that point to identical positions are grouped and taken as evidence of an *Alu *insertion ~20 bp from that point. At this point, nearly all read pairs fall into widely separated groups, each identified by a unique chromosomal position. Due to rare sequencing errors and alignment variations, a very few read pairs (< 2% of mapped read pairs) are positioned singly within 3 bp of positions supported by many read pairs. These were merged with the most numerous neighboring group. The result is a list of chromosomal positions that identify all *Alu *insertion loci retrieved by ME-Scan from these samples. The list of chromosomal positions and the number of paired reads supporting them, broken down by sample (experiment and index), constitutes the data from which the presence/absence genotype of a putative *Alu *insertion in a sample is judged.

### Annotation of Alu insertions identified by ME-Scan

We used BLAST and the RepeatMasker annotation of the reference genome (hg19/GRCh37) to annotate the loci identified by ME-Scan with information about known *Alu *elements. A local BLAST server (blastall 2.2.18, [59]; arguments: blastn, word size 7, gap open cost 50, gap extension cost 2, essentially forbidding alignment gaps) was used to identify all matches to the ALUSPv2 sequencing primer binding site (PBS) in the human reference genome (hg19/GRCh37, without the nine alternate haplotype chromosomes.) We then linked the sequencing PBS identified by BLAST to the RepeatMasker *Alu *elements that contain them. Nearly all (98.7%) of the sequencing PBS matches are contained in an annotated *Alu *element. We computed the position of the end of the 36-bp sequencing read that would be generated by ME-Scan from each sequencing PBS. That position should be identical to the chromosomal position identifier we used to label ME-Scan loci, so we linked the sequencing PBS and associated RepeatMasker information to the corresponding ME-Scan loci by comparing those two positions (with ±3 bp of tolerance to allow for small variations in mapping and BLAST alignments.) With a single exception, all of the sequencing PBS that matched ME-Scan loci in this way were also contained in RepeatMasker-annotated *Alu *insertions. The exception (on chromosome 9, at position 133,999,977) appears to be in a small duplicated fragment of an immediately adjacent *Alu*; it was labeled as *'Alu'*.

The annotation of ME-Scan loci with RepeatMasker information reveals three classes of loci: *AluYb8/9 *insertions present in the reference genome; other (mostly older) *Alu *elements also present in reference that amplified sporadically in ME-Scan, despite lacking perfect primer binding sites; and those that could not be annotated and presumably represent new *AluYb8/9 *insertions. To aid the analysis of new insertions, all *Alu *junction-end sequence reads were checked for the presence or absence of the expected *Alu *sequence. Reads that matched the expected *Alu *sequence at 10 bp or yielded a Smith-Waterman alignment score of ≥45 (gap opening cost of 64, gap extension cost of 8) when aligned to the expected 16 bp of *Alu *sequence (5' GCC ACC GCG CCC GGC 3') were identified as having the *Alu *sequence. If at least 50% of the junction reads supporting an *Alu *insertion locus contained detectable *Alu *sequence, then the locus itself was annotated as having the *Alu *sequence.

### Identification of a set of putatively fixed, retrievable Alu loci

We used BLAST [59] and dbRIP [16] to collect a set of *AluYb8/9 *loci that are likely to be present and retrievable in all the individuals we tested. All matches to the *AluYb8/9*-specific portion of the ME-Scan amplification primer (the 3' 20 bp of ALUBP2) were identified using BLAST, as above. We then selected loci that met the following criteria: amplification and primer binding sites were present, with BLAST HSP scores of at least 32.2 and 46.1 (respectively); their start positions were separated by 224 ± 15 bp; both PBS had the same orientation; the sequencing PBS was downstream (3') of the amplification PBS; and there were no mismatches to the corresponding primer in the 3' 10 bp of either PBS. We removed insertions that are known to be polymorphic by virtue of being listed in dbRIP [16]. 1,708 loci met these criteria (Additional File 3).

### Confirmation of insertions by PCR and gel electrophoresis

Unique PCR primers flanking *AluYb8/9 *loci were designed from the human genome reference sequence. Standard PCR and gel electrophoresis protocols [60] were used to amplify and visualize DNA fragments from each individual. *Alu *insertion genotypes were called only if the expected fragment sizes (which differ by ~300 bp, the size of a typical *Alu*) were clearly evident and free of other unexplained products. The genomic location, primer sequences, PCR conditions and genotyping results are given in Additional Files 4, 5, 6 and 7.

## Authors' contributions

DW conceived of the method, designed the biochemical and computational protocols, and wrote the manuscript. JX conceived of the sampling and pooling design, participated in the design and testing of the biochemical and computational protocols, and participated in writing the manuscript. YZ participated in the design and testing of the biochemical protocol, carried out the library preparations, assessed their quality, and generated confirmatory genotypes by PCR and gel electrophoresis. WW participated in the design of the biochemical protocol, and in assessment of library quality. LJ and MB participated in the design of this study, its coordination, and in manuscript preparation. All authors read and approved the final manuscript.

## Supplementary Material

Additional file 1 **List of 5,053 *Alu *insertion loci identified as well-supported by ME-Scan (see Methods.)**. Click here for file

Additional file 2 **Number of sequencing read pairs supporting each of 5,053 *Alu *insertion loci identified by ME-Scan, for each of seven samples**. Click here for file

Additional file 3 **List of 1,708 *Alu *insertion loci present in hg19/GRCh37 (without nine alternate haplotype chromosomes), absent from dbRIP, with good primer binding sites for our amplification and sequencing primers**. Click here for file

Additional file 4 **List of 69 *Alu *insertion loci genotyped by PCR and gel electrophoresis for this work**. Click here for file

Additional file 5 **List of 44 *Alu *insertion loci genotyped by PCR and gel electrophoresis in eight additional individuals (six unrelated individuals of east Asian descent **[42], **one African (NA19376 from Webuye, Kenya), and one European (J. C. Venter)**. Click here for file

Additional file 6 **Description of 38 *Alu *loci genotyped in previous work **[60]. Click here for file

Additional file 7 **Genotypes at 38 *Alu *insertion loci and 4 individuals, according to previous genotyping by PCR and gel electrophoresis **[60]. Click here for file
